# MiR-383-5p promotes schistosomiasis-induced liver fibrosis by targeting peroxiredoxin-3

**DOI:** 10.1186/s13071-025-06824-w

**Published:** 2025-06-03

**Authors:** Yi-Xin Li, Xin-Yue Zhang, Ju-Lu Lu, Ying-Ying Yang, Cong-Jin Mei, Pan-Pan Dong, Chuan-Xin Yu, Jian-Feng Zhang, Chun-Rong Xiong, Li-Jun Song, Kun Yang

**Affiliations:** National Health Commission Key Laboratory of Parasitic Disease Control and Prevention, Jiangsu Provincial Key Laboratory on Parasite and Vector Control Technology, Jiangsu Provincial Medical Key Laboratory, Jiangsu Institute of Parasitic Diseases, Wuxi, 214064 China

**Keywords:** miR-383-5p, PRDX3, ROS, Liver fibrosis, Schistosomiasis

## Abstract

**Background:**

Schistosomiasis-induced liver fibrosis, a major complication of infection, arises primarily from the host immune response to schistosome eggs. The mechanisms underlying the development of liver fibrosis remain unclear, but microRNAs (miRNAs) are thought to play a crucial role in this process. Our previous study revealed significantly reduced *miR-383-5p* expression in patients with advanced schistosomiasis, particularly in those with newly developed disease, suggesting a possible association between *miR-383-5p* and fibrotic progression. This study explores the role and mechanism of *miR-383-5p* in schistosomiasis-induced liver fibrosis.

**Methods:**

The target gene of *miR-383-5p* was predicted through bioinformatics analysis. The expression levels of *miR-383-5p* and its target gene in the livers of *Schistosoma japonicum (S. japonicum)*-infected mice were investigated. Dual-luciferase reporter assays and *miR-383-5p* mimics and inhibitors were transfected of into LX-2 cells to determine the regulation of *miR-383-5p* on its target gene. AAV-8-overexpressing *miR-383-5p* vector injected into mice infected with *S. japonicum*, the target gene expression level, fibrosis-related factors, and pathological changes of liver were evaluated. The target gene knockout mice were infected with *S. japonicum*, and the degree of liver fibrosis was detected.

**Results:**

Target gene prediction identified *peroxiredoxin 3* (*PRDX3*), a mitochondrial peroxidase that scavenges reactive oxygen species (ROS), as a target of *miR-383-5p*. During the progression of schistosome infection in mice, the expression level of *miR-383-5p* in the liver gradually decreased, reaching its lowest level 6 weeks after infection, at the peak of inflammation in egg granulomas, then gradually increasing, while the expression kinetics of *PRDX3* were opposite to those of *miR-383-5p*. Using dual-luciferase reporter assays and transfection of *miR-383-5p* mimics and inhibitors into LX-2 cells, we confirmed that *miR-383-5p* directly targeted the 3′ untranslated region (UTR) of *PRDX3*, leading to decreased mRNA levels of *PRDX3*. AAV8-mediated *miR-383-5p* overexpression and *PRDX3* knockout in the mice infected with *S. japonicum* led to increased hepatic ROS and promoted the schistosomiasis-induced liver fibrosis.

**Conclusions:**

Our findings suggest that downregulating *miR-383-5p* after schistosome infection may alleviate liver inflammation by de-repressing *PRDX3*, thereby increasing ROS scavenging and reducing oxidative stress. This study elucidates the role of the *miR-383-5p*/PRDX3 axis in schistosomiasis-induced liver fibrosis, suggesting that PRDX3 is a potential therapeutic target for this disease.

**Graphical abstract:**

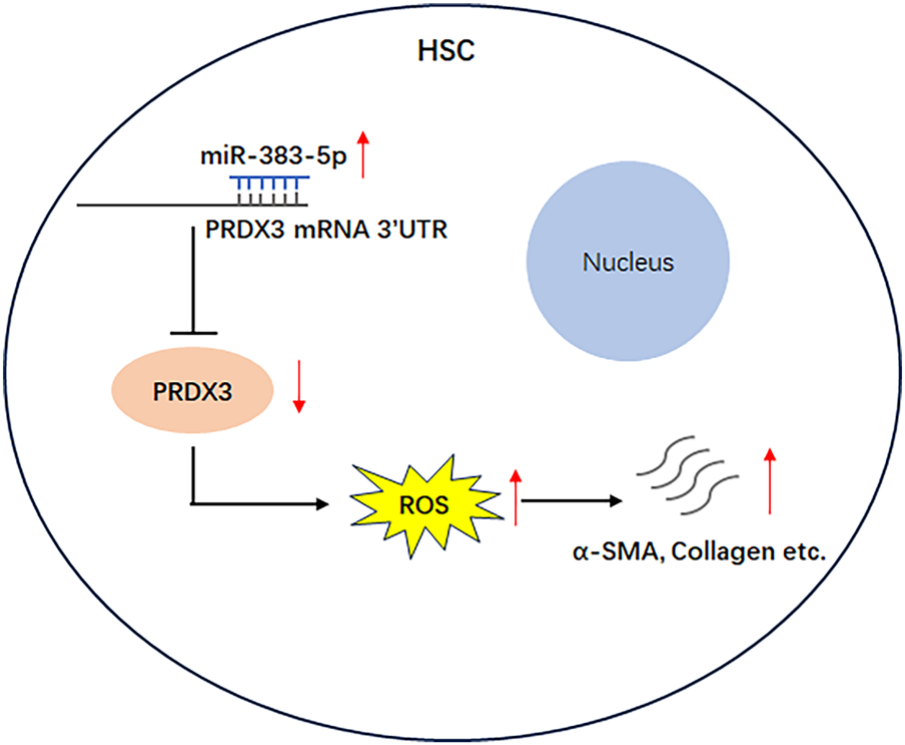

## Background

Schistosomiasis, a parasitic disease endemic to tropical and subtropical regions, remains a significant global health burden [1]. Current epidemiological data indicate approximately 230 million infections across 78 countries annually, with over 800 million people worldwide at risk of exposure. The disease is estimated to contribute to more than 200,000 deaths each year [2]. The primary pathological mechanism involves hepatic egg granuloma formation and subsequent liver fibrosis caused by parasite egg deposition [3]. Eggs retained in hepatic tissue trigger sustained host immune responses, initiating granulomatous inflammation that drives a cascade of pathophysiological changes. The chronic inflammatory process culminates in progressive fibrosis, ultimately manifesting as severe complications including portal hypertension, hepatosplenomegaly, and ascites, and in the advanced stages, cirrhosis and hepatocellular carcinoma [4, 5]. Notably, even following successful praziquantel (PZQ) therapy to eliminate adult worms, residual eggs continue to mature and release soluble egg antigen (SEA). This persistent antigen maintains granulomatous responses and fibrotic progression, potentially leading to newly developed advanced schistosomiasis cases despite anti-helminthic treatment [6]. This phenomenon is corroborated by epidemiological studies demonstrating continued disease progression in previously treated patients from transmission-controlled areas, with many ultimately developing newly developed advanced schistosomiasis [7].

The development of liver fibrosis in schistosomiasis involves multiple etiological factors, with the activation of hepatic stellate cells (HSCs) serving as a central mechanism. Activated HSCs drive fibrogenesis through excessive secretion of extracellular matrix (ECM) proteins, which accumulate in hepatic tissues. While the molecular pathways underlying HSC activation are complex, emerging evidence highlights microRNAs (miRNAs) as critical regulators in the development of liver fibrosis caused by schistosomiasis [8]. MiRNAs, a class of highly stable short non-coding RNAs, exert post-transcriptional control by binding to complementary sequences in the 3′ untranslated regions (3′ UTRs) of target mRNAs, leading to transcript degradation or translational repression. These molecules orchestrate diverse biological processes, including cell proliferation, migration, fibrotic remodelling, and immune modulation, through their pleiotropic gene regulatory functions [9–12]. Notably, dysregulated miRNA profiles have been conclusively linked to inflammatory and fibrotic pathologies in murine models of schistosome infection [11, 13–15].

Our previous work analysed miRNA expression in the peripheral blood samples from residents of historically endemic schistosomiasis regions. We found significant differences in the expression of circulating miRNAs between newly developed advanced schistosomiasis patients, PZQ-treated individuals (previously infected subjects who completed standardized therapy without meeting advanced schistosomiasis criteria), and healthy controls. Specifically, newly developed advanced schistosomiasis patients exhibited 4 upregulated and 16 downregulated circulating miRNAs relative to controls, while the PZQ-treated group demonstrated 6 upregulated and 21 downregulated miRNAs compared to healthy individuals [16]. Of particular interest, *miR-383-5p* displayed marked suppression, with expression levels reduced 4.23-fold (PZQ-treated) and 11.82-fold (newly developed advanced schistosomiasis) versus controls. The results revealed that the expression of *miR-383-5p* was significantly inhibited in patients with a history of treatment and newly developed advanced schistosomiasis, especially in newly developed advanced schistosomiasis patients, indicating that *miR-383-5p* may play a role in the progression of liver fibrosis caused by schistosome infection.

Bioinformatics analysis via the TargetScan database (http://www.targetscan.org/mmu_72/) identified *peroxiredoxin-3* (*PRDX3*), a thioredoxin-dependent mitochondrial antioxidant, as a principal *miR-383-5p* target [17, 18]. PRDX3, a phylogenetically conserved member of the peroxiredoxin family, regulates redox homeostasis by scavenging reactive oxygen species (ROS) and mitochondrial hydrogen peroxide (H_2_O_2_), thereby mitigating oxidative stress. Beyond its cytoprotective function, PRDX3 modulates signalling cascades governing cell differentiation, proliferation, and apoptosis [19–21].

In this study, our murine model of *Schistosoma japonicum (S.japonicum)* infection demonstrated an inverse correlation between hepatic *miR-383-5p* suppression and *PRDX3* upregulation during fibrotic pathogenesis. Functional studies confirmed that *miR-383-5p* negatively regulated *PRDX3* expression in vitro and in vivo. This regulatory axis potentiates oxidative stress within hepatic microenvironments, creating a permissive milieu for HSC activation and subsequent fibrogenesis. These discoveries not only elucidate a novel mechanism underlying liver fibrosis caused by schistosomiasis but also identify the *miR-383-5p*/PRDX3 pathway as a promising therapeutic target for antifibrotic interventions.

## Methods

### Animals

Female C57BL/6 mice (6‒8 weeks old, 15‒25 g, specific-pathogen-free) were obtained from the Animal Center of Yangzhou University. *S. japonicum*-infected snails were provided by the Jiangsu Institute of Parasitic Diseases (JIPD). PRDX3 heterozygous mice (PRDX3^±^) were purchased from Cyagen Biotechnology (Suzhou, China) and bred at the JIPD. Genotyping was performed via a mouse genotype identification kit (Vazyme, Nanjing, China). All animal experiments were approved by the Ethics Review Committee of the Jiangsu Institute of Parasitic Diseases (approval ID: JIPD-2022–003).

### Cell culture

A total of 293 T cells and LX-2 cells were purchased from Pricella Biotechnology Co., Ltd. (Wuhan, China) and cultured in Dulbecco’s modified Eagle medium (DMEM) supplemented with 10% foetal bovine serum at 37 °C in a humidified incubator containing 5% CO_2_.

### Establishment of a mouse model infected with *S. japonicum*

A cohort of 29 female C57BL/6 mice were percutaneously infected with 15 ± 1 *S. japonicum* cercariae and sacrificed at 3, 6, 9, and 12 weeks post-infection (*n* = 6 per time point). Within this experimental design, a group of infected mice (*n* = 5) were treated with PZQ (250 mg/kg/day) via oral gavage for three consecutive days at the 9-week post-infection time point, followed by terminal sacrifice at 12 weeks post-infection. Another five uninfected mice were used as the control group (0-week time point). Liver tissues of each mouse were collected for subsequent histopathological and molecular analyses after sacrifice.

### AAV-8-overexpressing *miR-383-5p* vector was injected into mice infected with *S. japonicum*

The AAV8 vector carrying *miR-383-5p* (AAV-8-overexpressing *miR-383-5p* vector) and the AAV8 vector were synthesized by Jiman Biotechnology Co., Ltd. (Shanghai, China). C57BL/6 mice, 5 to 6 weeks old, were divided into four groups, with six mice in each group: the uninfected control group, the infected PBS injection group, the infected AAV8 empty vector injection group, and the infected AAV8-miR-383-5p injection group. After 10 days post-infection, each mouse in the experimental group was injected with the AAV8 vector carrying *miR-383-5p* via the tail vein at a titre of 10^12^ VG in 100 μl. The other two infection groups were injected with equal amounts of phosphate-buffered saline (PBS) or the AAV8 empty vector as controls. Mice were sacrificed on day 50 post-infection. The livers were immediately harvested from all experimental subjects for further study.

### Dual-luciferase reporter gene assay

Mimics of *miR-383-5p* and their control sequences were synthesized by Sagan Corporation (Shanghai, China). The reporter gene plasmid pmirGLO-PRDX3-WT and its mutants were synthesized by Jiman Biotechnology Co., Ltd. (Shanghai, China). A total of 500 ng of pmirGLO-PRDX3-WT or mutants with 500 ng of *miR-383-5p* mimics or negative controls (NC) were transfected into 293 T cells with Lipofectamine 3000 (Invitrogen, CA, USA). Firefly luciferase activity and Renilla luciferase reporter gene activity were determined via a dual-luciferase reporter gene assay kit (Vazyme, Nanjing, China) 48 h after transfection, and the ratio of firefly luciferase activity to Renilla luciferase activity was used as the final detection result. Each group was repeated three times.

### Transfection of *miR-383-5p* mimics and inhibitors into LX-2 cells

The mimics, inhibitors, and control sequences of *miR-383-5p* were synthesized by Sagan Corporation (Shanghai, China). A total of 1 × 10^6^ LX-2 cells/well were cultured in a six-well plate, and then 2500 ng of *miR-383-5p* mimics/inhibitors or corresponding NC mimics/inhibitors were transfected into LX-2 cells with Lipofectamine™ 3000 (Invitrogen) according to the manufacturer’s instructions, which were cultured for 48 h before the cells were collected for activity and ROS detection. The sequences of *miR-383-5p* mimic and inhibitor are shown in Table 1.

**Table 1:** Mimic and inhibitor sequences of *miR-383-5p*

	Sequences
Mimic of *miR-383-5p*	AGAUCAGAAGGUGAUUGUGGCU CCACAAUCACCUUCUGAUCUUU
Inhibitor of *miR-383-5p*	AGCCACAAUCACCUUCUGAUCU

### Quantitative reverse transcription polymerase chain reaction (qRT-PCR)

RNA from 10^6^ cells or 100 mg of liver tissue was extracted via the TRIzol method and reverse transcribed into cDNA with a first-strand cDNA synthesis kit (Vazyme, Nanjing, China). PCRs were performed with a fluorescence quantitative PCR kit (SYBR Green) (Vazyme, Nanjing, China), and the primers used are shown in Table 2. The reaction conditions were as follows: 95 °C for 30 s; 95 °C for 5 s, 55 °C for 15 s, and 72 °C for 10 s; 40 cycles. Relative expression levels were calculated via the 2^−ΔΔCt^ method, with *GAPDH* used as a housekeeping gene.

**Table 2:** qPCR primer sequences

	Sequences
*Collagen I*	Forward: GCTCCTCTTAGGGGCCACT Reverse: ATTGGGGACCCTTAGGCCAT
*Collagen III*	Forward: CTGTAACATGGAAACTGGGGAAA Reverse: CCATAGCTGAACTGAAAACCACC
*α-SMA*	Forward: CCCAGACATCAGGGAGTAATGG Reverse: TCTATCGGATACTTCAGCGTCA
*TGF-β*	Forward: CCACCTCAAGACCATCGAC Reverse: CTGGCGAGCCTTAGTTTGGAC
*MMP-9*	Forward: GCAGAGGCATACTTGTACCG Reverse: TGATGTTATGATGGTCCCACTTG
*TIMP-1*	Forward: CGAGACCACCTTATACCAGCG Reverse: ATGACTGGGGTGTAGGCGTA
*PRDX3*	Forward: GGTTGCTCGTCATGCAAGTG Reverse: CCACAGTATGTCTGTCAAACAGG
*GAPDH*	Forward: TGGAAGATGGTGATGGGATT Reverse: TCAACGGATTTGGTCGTATTG
*miR-383-5p* stem-loop	GTCGTATCCAGTGCAGGGTCCGAGGTATTCGCACTGGATACGACAGCCAC
*miR-383-5p*	Forward: GCGCGAGATCAGAAGGTGACT Reverse: AGTGCAGGGTCCGAGGTATT
*U6*	Forward: CTCGCTTCGGCAGCACA Reverse: AACGCTTCACGAATTTGCGT

### Western blot (WB) analysis

One millilitre of RIPA lysis buffer with protease inhibitor and phosphatase inhibitor was added to 100 mg of liver tissue. The mixture was centrifuged at 12,000 rpm (12,830×*g*) at 4 °C for 5 min, and the protein concentration of the supernatant was detected via the bicinchoninic acid (BCA) method. A total of 30–100 μg of protein was separated via sodium dodecyl sulfate–polyacrylamide gel electrophoresis (SDS‒PAGE), transferred onto a polyvinylidene fluoride (PVDF) membrane with a semi-dry transfer instrument (Bio-Rad, USA), and then incubated with anti-collagen I and α-smooth muscle actin (α-SMA) antibodies (1:1000 dilution, CST, USA) overnight at 4 °C. Then, the samples were incubated with sheep anti-rabbit antibody (1:4000 dilution, Jackson, USA) at room temperature for 1 h, followed by enhanced chemiluminescence (ECL) staining. Three to four biological replicates of each group were used in the experiment, and the expression relative to GAPDH was analysed using ImageJ software.

### Hematoxylin and eosin (HE) and Masson staining

Liver tissue was fixed in 4% paraformaldehyde and routinely dehydrated and embedded. Slices with a thickness of 4 µm were stained via HE and Masson detection kits from Solarbio Biotechnology Co., Ltd. (Beijing, China). In HE staining slices, all of the single egg granulomas in the 10 ± 2 slices from each group were photographed with CellSense under a microscope (Olympus, Japan), and the areas of single egg granulomas were calculated using cellSens (Olympus, Japan). In Masson staining slices, a total of 10 ± 2 slices from each group were photographed with CellSense under a microscope (Olympus, Japan), areas across the entire field of view were taken, and the percentage of positive staining area was measured using ImageJ software.

### ROS analysis

The ROS levels in LX-2 cells were determined with a 2′,7′-Dichlorodihydrofluorescein diacetate (DCFH-DA) kit (BioLegend, San Diego, CA, USA) to measure the fluorescence intensity via flow cytometry. A total of 1 × 10^6^ LX-2 cells in 1 ml PBS were incubated with 10 μM DCFH-DA for 20 min at 37 °C in a humidified incubator containing 5% CO_2_. Cells were washed three times with PBS, then the fluorescence intensity was detected by flow cytometry (Beckman, CA, USA) using a 488-nm excitation wavelength and 525-nm emission wavelength. The ROS analysis in 100 mg of liver tissue was carried out via a reagent kit (Bestbio, Shanghai, China) according to the manufacturer’s instructions. The intensity of the ROS in the liver tissue was calculated as the fluorescence intensity (relative fluorescence units (RFU)]/protein concentration (mg protein).

### Statistical analysis

One-way analysis of variance (ANOVA) was used for comparisons among multiple groups, and a *t*-test was used for comparisons between groups. GraphPad Prism 9.0 was used for plotting, and SPSS Statistics 25 was used for statistical analysis. A significance level of *P* < 0.05 was considered statistically significant.

## Results

### The expression of *miR-383-5p* and *PRDX3* in the liver of mice infected with schistosome

**Fig. 1:**
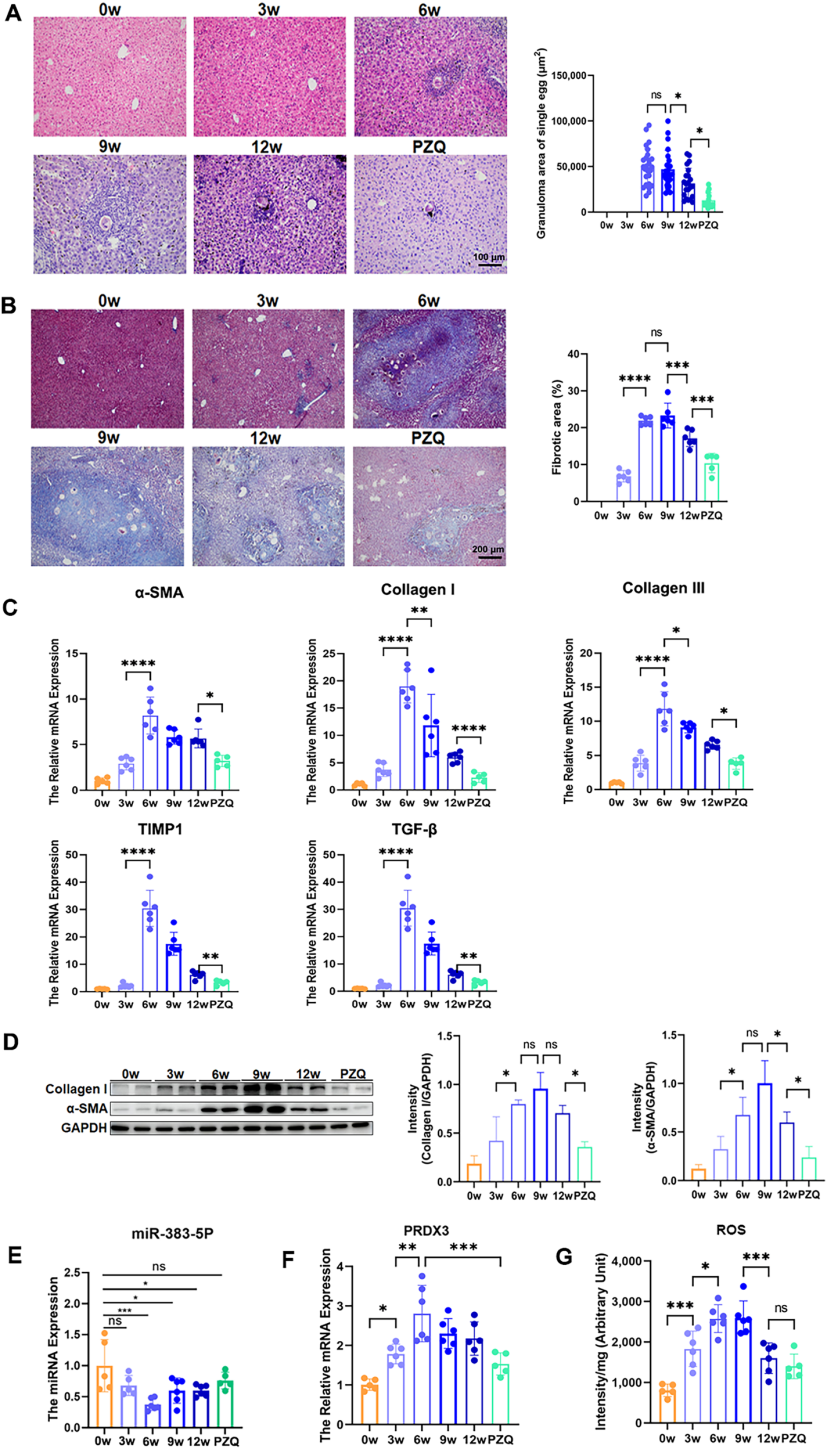
The *miR-383-5p* or *PRDX3* expression in the mice infected with schistosome. **A** Area of the single egg granuloma in the mouse liver at different stages of infection (HE staining, ratio = 200 µm) and statistical analysis. **B** Percentage of fibrotic area in the liver of the mice at different stages of infection (Masson staining, ratio = 200 µm) and statistical analysis. **C** The mRNA expression level of *α-SMA*, *collagen* *I*, *collagen* *III*, and *TGF-β* in the livers of mice at different stages of schistosome infection. **D** The protein expression levels of α-SMA and collagen I in the livers of mice infected with schistosomes at different stages. **E** The miRNA expression level of *miR-383-5p* in the livers of mice infected with schistosomes at different stages. **F** The mRNA expression level of *PRDX3* in the livers of mice infected with schistosomes at different stages. **G** Levels of ROS in the livers of mice infected with schistosomes at different stages. * *P* < 0.05, **P < 0.01, ***P < 0.001, ****P < 0.0001

HE staining revealed that the average areas of single egg granulomas at 6, 9, and 12 weeks and with PZQ after schistosome infection were 49,255.85 ± 20,187.77, 47,069.91 ± 20,641.94, 31,618.45 ± 16,416.56, and 13,094.94 ± 7973.47 μm^2^, respectively. No obvious egg granulomas were observed at 3 weeks after schistosome infection, and the area of the single egg granulomas in the liver was the largest at 6 weeks after infection. The area decreased slightly at 9 weeks and 12 weeks after infection. Compared with the infection group, the PZQ treatment group presented a significant decrease in area (*F* = 16.98, *P* < 0.05) (Fig. 1A). The Masson staining results revealed that fibrotic area proportion at 3, 6, 9, and 12 weeks, and with PZQ after schistosome infection were 6.82 ± 1.55%, 21.89 ± 1.17%, 23.30 ± 3.36%, 17.13 ± 2.25%, and 10.39 ± 2.65%, respectively. No significant fibrosis was detected in the liver at 3 weeks after schistosome infection, whereas fibrotic area proportion was the largest at 9 weeks and decreased after 12 weeks. The fibrotic area in the PZQ treatment group was significantly reduced (*F* = 55.78, *P* < 0.05) (Fig. 1B). The mRNA expression levels of the fibrosis-related molecules* α-SMA*, *collagen I*, *collagen III*, *tissue inhibitor of metalloproteinase 1* (*TIMP1*), and *transforming growth factor beta* (*TGF-β*) in the livers of the mice at different stages of schistosome infection were determined via qRT-PCR. The results revealed that the mRNA levels of these factors gradually increased after infection, reached their highest level at 6 weeks, and then gradually decreased, whereas the expression levels in the PZQ treatment group decreased significantly (*F* = 19.47, 29.25, 35.95, 63.81, 46.01, respectively; *P* < 0.05) (Fig. 1C). The WB results revealed that the protein expression of α-SMA and collagen I gradually increased after infection, peaked at 9 weeks, and then decreased at 12 weeks after infection but remained significantly greater than that in the uninfected group, with a significant decrease in the PZQ treatment group (*F* = 14.56, 13.04, *P* < 0.05) (Fig. 1D). The miRNA expression level of *miR-383-5p* was determined via qRT-PCR (with *U6* as the housekeeping gene), and the results revealed that the miRNA expression level of *miR-383-5p* gradually decreased after infection, with the lowest expression level occurring at 6 weeks after infection. During the subsequent infection process, the expression level slightly increased but was still significantly lower than that in the control group (*F* = 11.347, *P* < 0.05) (Fig. 1E). The mRNA expression level of *PRDX3* gradually increased after infection, peaked at 6 weeks, and then gradually decreased (*F* = 12.53, *P* < 0.05) and was negatively correlated with the *miR-383-5p* level (Fig. 1F). The ROS level in the liver of mice infected with schistosome gradually increased after infection, peaked at 9 weeks, and then gradually decreased (*F* = 20.84, *P* < 0.05) (Fig. 1G).

### *MiR-383-5p* targets *PRDX3* and downregulates its expression

**Fig. 2:**
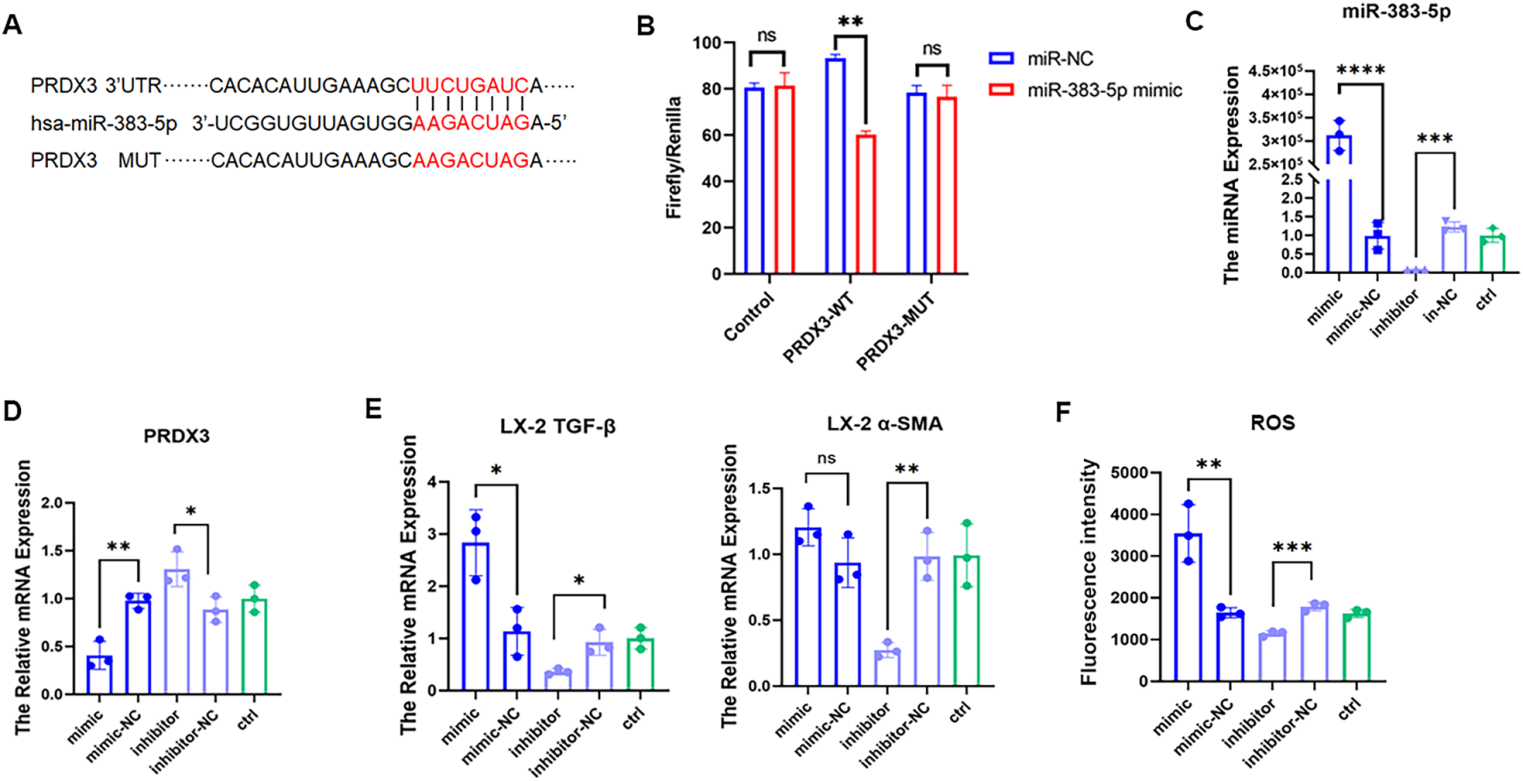
*MiR-383-5p* targeted *PRDX3* and downregulated its expression. **A** Sequence alignment of *miR-383-5p* and the complementary region of the 3′UTR of *PRDX3*. **B** Fluorescent enzyme activity detection of *miR-383-5p* binding to the 3′ UTR of *PRDX3*. **C** The miRNA expression level of *miR-383-5p* in LX-2 cells transfected with *miR-383-5p* mimics or inhibitors. **D** The mRNA expression level of *PRDX3* in LX-2 cells transfected with *miR-383-5p* mimics or inhibitors. **E** The mRNA expression level of *α-SMA* and *TGF-β* in LX-2 cells transfected with *miR-383-5p* mimics or inhibitors. **F** ROS fluorescence intensity in LX-2 cells transfected with *miR-383-5p* mimics or inhibitors. *P < 0.05, **P < 0.01, ***P < 0.001, ****P < 0.0001

The binding site of *miR-383-5p* and the 3’ UTR region of *PRDX3* is shown in Fig. 2A. *MiR-383-5p* mimics or the control (NC) and empty reporter gene vectors were co-transfected into 293 T cells, and there was no significant difference in luciferase activity between the *miR-383–5p* mimics and the control (NC) groups (*t* = 1.395, *P* > 0.05). After co-transfection with *miR-383-5p* NC or mimic and *PRDX3*-UTR vectors, the luciferase activity of the *miR-383-5p* mimics was significantly inhibited (*t* = 5.436, *P* < 0.05) (Fig. 2B). These results suggest that *miR-383-5p* can bind to *PRDX3*, confirming that *PRDX3* is the target gene of *miR-383-5p*. The *miR-383-5p* mimics or inhibitors were transfected into LX-2 cells, and the miRNA expression level of *miR-383-5p* in the cells was determined via qRT-PCR (*U6* as the housekeeping gene). The results revealed that the miRNA expression level of *miR-383-5p* was significantly increased in the mimic group (t = 14.69, *P* < 0.0001) and decreased in the inhibitor group (*t* = 13.04, *P* < 0.001), indicating that the transfection was successful (Fig. 2C). qRT-PCR was used to determine the mRNA expression level of *PRDX3* in LX-2 cells. Compared with that in the control group, the mRNA expression of *PRDX3* in the mimic group decreased (*t* = 5.921, *P* < 0.01), whereas the expression in the inhibitor group increased (*t* = 3.235, *P* < 0.05) (Fig. 2D). This finding was negatively correlated with the expression level of the *miR-383-5p* gene, confirming that *miR-383-5p* could negatively regulate the mRNA expression of *PRDX3* in LX-2 cells. The expression levels of the cell activation markers *α-SMA* and *TGF-β* were determined via qRT-PCR. The results revealed that the mRNA expression level of *α-SMA* in the mimic group increased, but the difference was not significant (*t* = 1.983, *P* > 0.05). The mRNA expression level of *TGF-β* significantly increased (*t* = 3.770, *P* < 0.05), whereas the mRNA expression levels of *α-SMA* and *TGF-β* in the inhibitor group decreased significantly (*t* = 6.493, 3.825, *P* < 0.05) (Fig. 2E). These results confirmed that *miR-383-5p* regulates the activation of LX-2 cells and promotes the expression of *α-SMA* and *TGF-β*. The fluorescence intensity of ROS in LX-2 cells was determined via flow cytometry. The fluorescence intensity of ROS in the mimic group of LX-2 cells increased significantly (*t* = 4.710, *P* < 0.01), whereas the fluorescence intensity of ROS in the inhibitor group decreased significantly (*t* = 9.221, *P* < 0.001) (Fig. 2F). These results indicated that *miR-383-5p* increased the level of ROS in LX-2 cells. After LX-2 cells were transfected with *miR-383-5p* mimics or inhibitors, the mRNA expression of *PRDX3* decreased or increased, and the level of ROS in the liver increased or decreased accordingly. These results confirmed that *miR-383-5p* could bind to *PRDX3* to regulate its expression.

### Overexpression of *miR-383-5p* downregulates *PRDX3* expression in vivo and increases the degree of liver fibrosis in mice infected with schistosomes

**Fig. 3:**
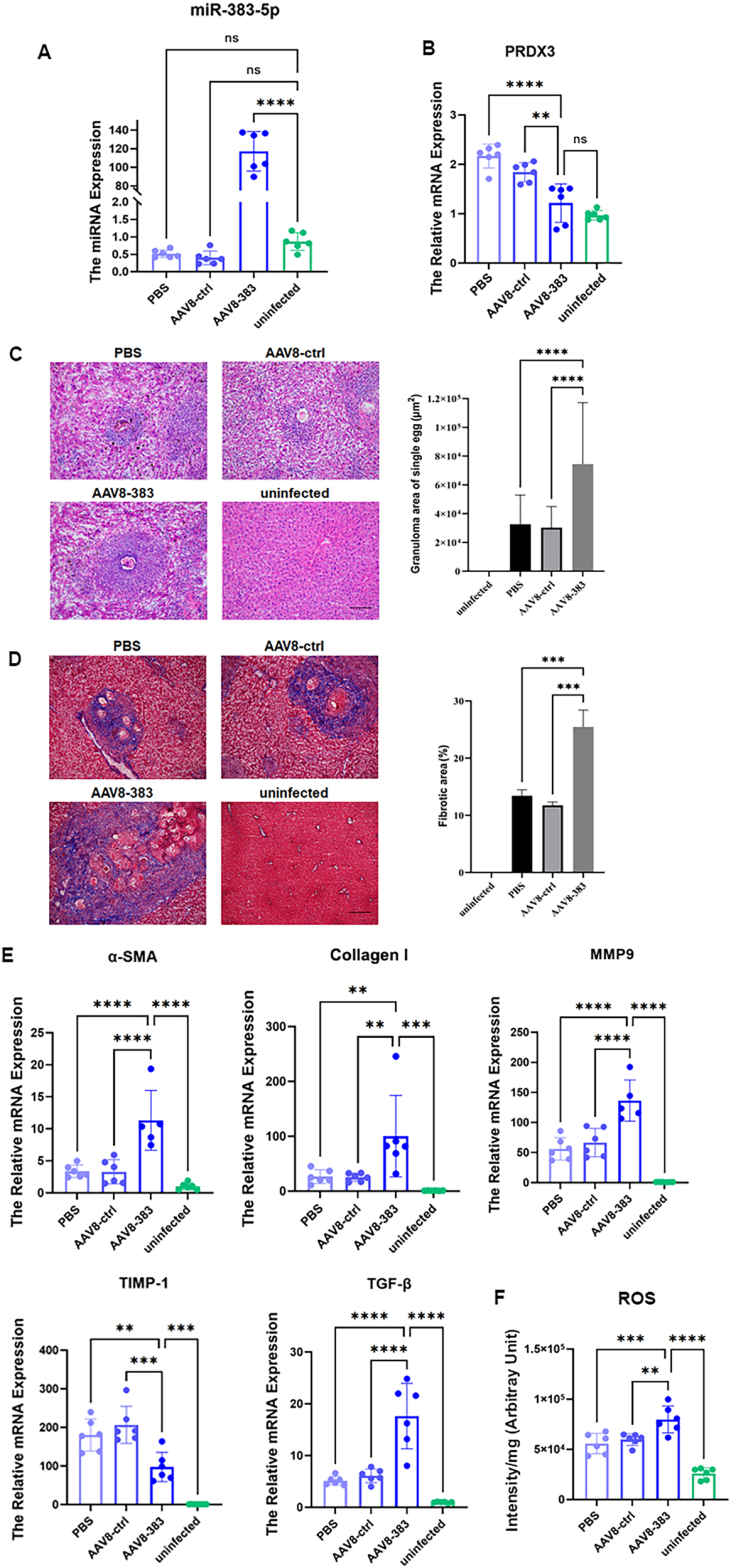
The degree of liver lesions in mice infected with schistosomes injected with the mmu-miR-383-5p-AAV8 overexpression plasmid. **A** The miRNA expression of *miR-383-5p* in the livers of mice infected with schistosomes. **B** The mRNA expression level of *PRDX3* in the livers of mice infected with schistosomes. **C** Area of single egg granulomas in the mouse liver (HE staining) and statistical analysis. **D** Percentage of fibrotic area in the livers of mice (Masson staining) and statistical analysis. **E** The mRNA expression level of *α-SMA*, *collagen I*, *MMP9*, *TIMP1*, and *TGF-β* in the livers of mice infected with schistosomes. **F** *MiR-383-5p* increased the level of ROS in the livers of mice infected with schistosomes. * *P* < 0.05, **P < 0.01, ***P < 0.001, ****P < 0.0001

AAV8-miR-383-5p was injected into the tail vein of mice infected with schistosomes. Compared with those in the PBS-injected group and the AAV8 empty vector control group, *miR-383-5p *expression was significantly increased (*F* = 179.8, *P* < 0.05) (Fig. 3A), and the mRNA level of *PRDX3 *was decreased in the AAV8-miR-383-5p group (*F* = 28.61, *P* < 0.05) (Fig. 3B). The above results suggested that the overexpression of *miR-383-5p* inhibited the mRNA expression of *PRDX3 *in the mouse liver. Compared with that in the PBS group and the AAV8 empty vector group, the area of a single egg granuloma in the liver was greater in the AAV8-miR-383-5p group, as shown by HE staining (*F* = 15.03, *P* < 0.05) (Fig. 3C). Compared with that in the PBS group and the AAV8 empty vector group, the fibrotic area proportion in the liver was greater in the AAV8-miR-383-5p group, as shown by Masson staining (*F* = 49.84, *P* < 0.05) (Fig. 3D), indicating that the overexpression of *miR-383-5p* in the mouse liver promoted the development of liver fibrosis caused by schistosomiasis. The mRNA expression levels of fibrosis-related factors (*α-SMA*, *collagen I*, *collagen III*, *MMP9*, *TIMP1*, and* TGF-β*) in mouse liver tissue were determined via qRT-PCR. Compared with those in the PBS group and the AAV8 empty vector control group, the mRNA expression levels of *α-SMA*, *collagen I*, *MMP9*, and *TGF-β* in the livers of the AAV8-miR-383-5p group were significantly greater (*F* = 18.30, 7.729, 43.50, 28.76, respectively; *P* < 0.05), whereas the expression of *TIMP1* was lower (*F* = 43.24, *P* < 0.05) (Fig. 3E). Compared with those in the PBS and AAV8 empty vector groups, the ROS levels in the livers of the mice in the AAV8-miR-383-5p group were greater (*F* = 33.73, *P* < 0.05) (Fig. 3F), indicating that the overexpression of *miR-383-5p* in the liver led to an increase in the ROS level, which is important for promoting fibrosis.

### *PRDX3* knockout increased the degree of liver fibrosis in mice infected with schistosomes

**Fig. 4:**
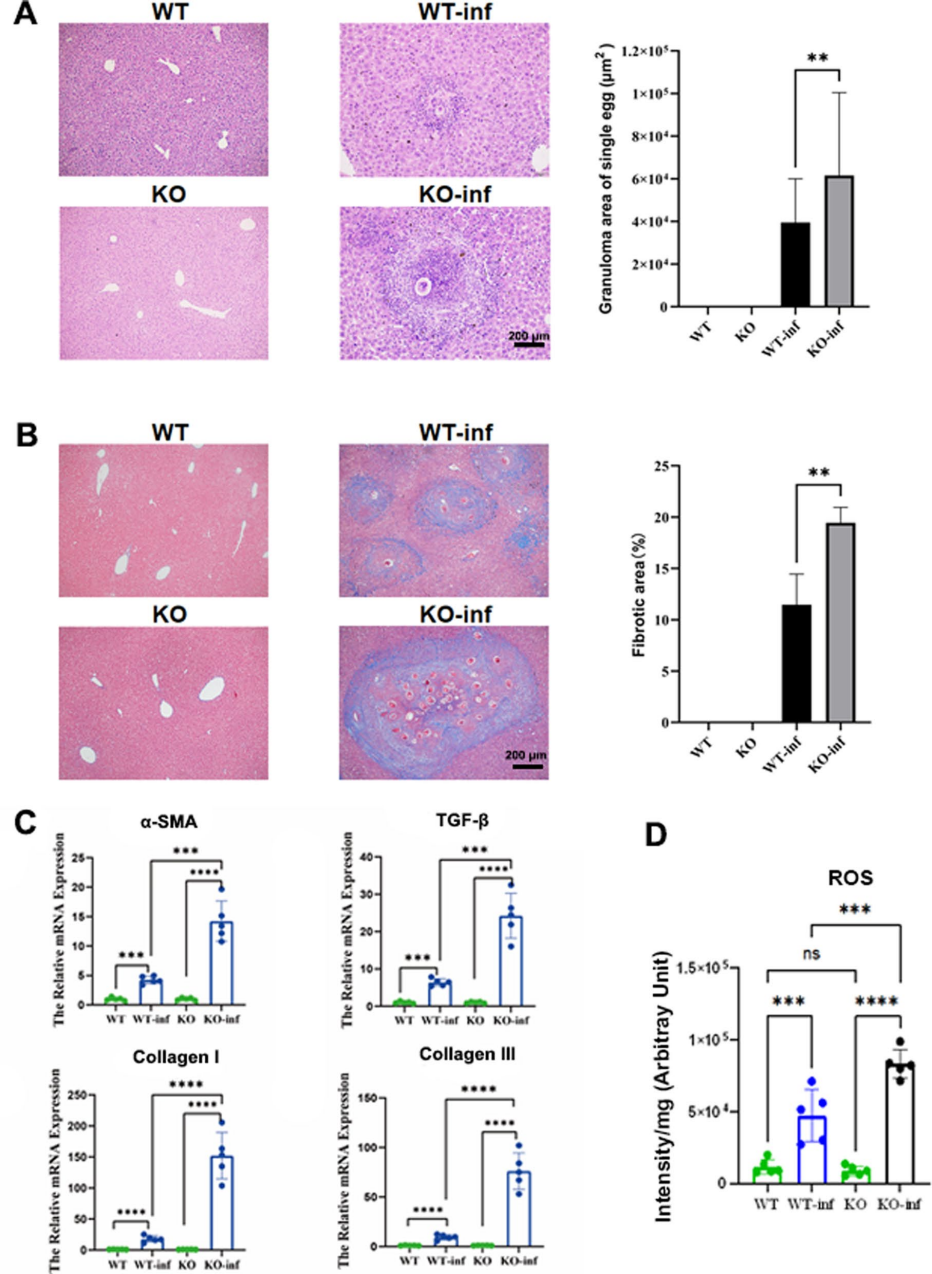
Liver fibrotic lesions are exacerbated in *PRDX3* knockout mice infected with schistosomes. **A** Pathological sections of liver granulomas (HE staining) and the area of a single egg granuloma in WT and *PRDX3* knockout mice. **B** Pathological sections of liver fibrosis (Masson staining) in WT and *PRDX3* knockout mice and the fibrotic area percentage. **C** The mRNA expression levels of *α-SMA*, *TGF-β*, *collagen I*, and *collagen III* in the livers of WT and *PRDX3* knockout mice. **D** Knockout of *PRDX3* increased ROS levels in the livers of schistosome-infected mice. * *P* < 0.05, **P < 0.01, ***P < 0.001, ****P < 0.0001

The HE and Masson staining results of liver pathological sections revealed that the areas of a single egg granuloma and collagen fibrotic area proportion in the liver increased significantly in the *PRDX3* knockout mice infected with schistosomes (*t* = 2.819, 4.178, *P* < 0.05) (Fig. 4A, B). The mRNA levels of *α-SMA*, *TGF-β*, *collagen I*, and *collagen III* in the livers of the *PRDX3* knockout mice were significantly increased (*t* = 6.400, 7.936, 8.042, and 6.3537, respectively; * P* < 0.05) (Fig. 4C). These results suggest that *PRDX3* knockout increases the degree of liver fibrosis in mice infected with schistosomes. Compared with those in the wild-type (WT) mice, ROS levels were increased in the livers of the *PRDX3* knockout mice infected with schistosomes (*F* = 51.36, *P* < 0.05) (Fig. 4D).

## Discussion

The eggs deposited in the liver induce a host immune response, leading to hepatic egg granuloma inflammation and subsequent liver fibrosis, which is the main pathological damage caused by schistosome infection in the host [22]. However, the mechanism of liver fibrosis caused by schistosome infection is still unclear, and effective treatment methods in the clinic are lacking [23]. Understanding the interaction between schistosomes and hosts is helpful for elucidating the molecular mechanism of liver fibrosis caused by schistosomiasis and discovering potential drug targets against liver fibrosis. Our previous studies revealed that the expression of *miR-383-5p* was significantly inhibited in patients with a history of treatment and newly developed advanced schistosomiasis, with a more pronounced decrease in newly developed advanced schistosomiasis patients, which suggested that *miR-383-5p* may play an important role in the progression of liver fibrosis caused by schistosome infection. In our study, we further confirmed that *miR-383-5p* directly targeted the 3′ UTR of *PRDX3*, leading to decreased PRDX3 protein levels. Overexpression of *miR-383-5p* in vivo promoted liver fibrosis in schistosomiasis.

During the process of schistosome infection in mice, the expression of *miR-383-5p* in the liver first decreased in the acute stage (6 weeks after infection) but gradually increased in the chronic (9 weeks after infection) and late stages of infection (12 weeks after infection) in mice infected with *S. japonicum*. However, the expression of *miR-383-5p* was lower than that in healthy mice. After PZQ treatment, the expression of *miR-383-5p* increased, but the level of *miR-383-5p* did not differ from mice in the uninfected group. The results are similar to the results of peripheral blood testing in the population, suggesting that the downregulation of circulating *miR-383-5p* expression may be related to the development of liver fibrosis caused by schistosomiasis. To further confirm this hypothesis, we identified collagen and pathological changes in the liver during the acute-stage, chronic-stage, late-stage, and PZQ treatment groups. The mRNA expression of *α-SMA* and *collagen I*, which are the most commonly used as indicators for HSC activation have been reported in the literature [24–26], and the area of egg granuloma in the mouse liver peaked at 6 weeks after infection and then decreased at 9 and 12 weeks, while the protein expression of α-SMA, collagen I, and the fibrosis area peaked at 9 weeks after infection of the late stage, which was consistent with previous reports [27–29]. Interestingly, the mRNA expression of *α-SMA *and *collagen I* were not inconsistent with the protein, as has also been reported in previous studies [30], which may be related to mRNA stability and post-transcriptional regulation [31].

These results revealed that the expression level of *miR-383-5p* in the liver gradually decreased, reaching its lowest level at 6 weeks after infection, at the peak of inflammation in egg granulomas, while the expression kinetics of *PRDX3* were opposite to those of *miR-383-5p* with the progression of inflammation in egg granulomas. The increased PRDX3 expression in livers infected with *S. japonicum* is to scavenge excess ROS deposition. These results suggest that during the development of inflammation, the host body may regulate the inflammatory response of egg granulomas by downregulating the expression of *miR-383-5p* to release the inhibitory effect on *PRDX3*, increasing its expression levels and the ability to scavenge ROS, ultimately reducing inflammation. This regulatory phenomenon may represent an evolutionarily conserved host defence mechanism to counteract pathogen-induced inflammatory cascades. Subsequently, the increased level of *miR-383-5p* gradually enhanced its transcriptional repression of *PRDX3*, resulting in diminished PRDX3 expression. This suppression precipitated intracellular ROS accumulation, thereby establishing a pro-fibrotic microenvironment conducive to liver fibrosis—pathological progression in chronic schistosomiasis. These results are in agreement with the finding of Xing et al. that IFN-γ can bind to the promoter region of *pre-miR-351* through STAT1 and IFN regulatory factor 2, negatively regulating the expression of *miR-351* at the early stage of schistosome infection (32 and 42 days post-infection). As infection progresses, the expression level of *miR-351* increases, and the inhibitory effect of vitamin D receptor (VDR) on the Smad signalling pathway is released via targeting of *VDR*, promoting the development of liver fibrosis [11]. These findings suggest that host miRNA downregulation may serve as a dual-edged immunomodulatory strategy: transient suppression enhances pathogen defences during acute infection stages, while sustained repression prevents immunopathology in chronic disease states. Such bidirectional miRNA modulation may establish a dynamic equilibrium, critically balancing pathogen clearance with host tissue preservation during chronic infection. The dynamic miRNA regulation has also been found in *Micrococcus luteus* infection, implying that dynamic miRNA expression may be essential for maintaining host immune homeostasis [32].

To explore the molecular mechanism of *miR-383-5p* regulating liver fibrosis caused by schistosomiasis, it was confirmed that *miR-383-5p* targeted the 3′ UTR of *PRDX3* through bioinformatics target gene prediction, dual-luciferase reporter gene, and transfection experiments. The results are consistent with that previous reported in the medulloblastoma [33]. Studies have shown that PRDX3 can scavenge ROS and H_2_O_2_ in cells, thereby reducing oxidative stress damage [19–21], indicating that PRDX3 exerts a protective effect against cellular oxidative stress. Oxidative stress is caused by an imbalance between the antioxidant system’s ability to scavenge ROS and the production of ROS, leading to an excess of ROS in cells and causing cellular dysfunction. Many studies have confirmed that oxidative stress is an important factor that promotes liver fibrosis [34], and excessive ROS can interfere with the function of liver cells, playing an important role in promoting the pathological process of liver fibrosis [35]. Oxidative stress can be detected in other liver fibrosis models [36], and the expression of *PRDX3* and amount of ROS in the livers of mice infected with *S. japonicum* were significantly increased in our study. Therefore, we believe that *miR-383-5p* plays a role in schistosomiasis-liver fibrosis by regulating the expression of *PRDX3* and affecting the oxidative stress of HSCs. This assumption was confirmed via overexpression of *miR-383-5p* using the AAV8 vector system in mice, which downregulated *PRDX3* expression, leading to elevated hepatic ROS levels and subsequent promotion of liver fibrosis. Additionally, knockout mice were used to confirm that PRDX3 alleviates liver fibrosis induced by schistosomiasis through scavenging ROS. The results revealed that *PRDX3* knockout increased the mRNA expression levels of the fibrosis-related factors *α-SMA*,* collagen I*, *collagen III*, and *TGF-β*, indicating that HSCs were activated to release the extracellular matrix. A significant increase in the area of single egg granulomas and surrounding collagen fibres suggested an exacerbation of liver fibrosis in *PRDX3* knockout mice. In addition, the level of ROS in the livers of *PRDX3* knockout mice was increased. The above results further confirmed that the PRDX3-mediated redox balance is an important mechanism for scavenging ROS and decreasing inflammatory responses in the host. The results were consistent with a previous report in liver fibrosis induced by CCl_4_ [37]. PRDX3 silencing exacerbates liver fibrosis and HSC activation, while HSC overexpression of PRDX3 alleviates liver fibrosis caused by CCl_4_ by inhibiting HSC activation through the mitochondrial ROS/TGF-β1/Smad2/3 signalling pathway [37].

The level of ROS in the livers of the mice infected with *S. japonicum* was similarly highest at 6 and 9 weeks, while the level of *PRDX3* reached its peak at 6 weeks. The results revealed that although *PRDX3* was upregulated after schistosome infection, significantly increased levels of ROS were detected in the liver. Upregulated expression of PRDX3 has been observed in colon cancer stem cells [38], prostate cancer cells [39], hepatocellular carcinoma [40], medulloblastoma [33], and breast cancer cells [41]. Similar to cancer cells, although *PRDX3* is upregulated after schistosome infection, the tissue still exhibits significantly high levels of ROS [42]. This phenomenon parallels findings in cancer research, suggesting that schistosome infection resulted in ROS production. Upon sensing oxidative stress, cells may upregulate *PRDX3* transcription through modulation of *miR-383-5p* expression. This enzyme subsequently catalyses H_2_O_2_ reduction, thereby enabling cellular defence against oxidative stress [43]. These findings suggest a potential homeostatic relationship between PRDX3 activity and ROS levels during schistosomiasis infection, though the precise regulatory mechanisms require further investigation.

## Conclusions

This study demonstrated that *miR-383-5p* negatively regulates *PRDX3* expression in* S. japonicum*-infected mice, contributing to exacerbated liver fibrosis. We confirmed that *miR-383-5p* directly targeted the 3′ UTR of *PRDX3*, leading to decreased mRNA expression levels of *PRDX3*. Overexpression of *miR-383-5p* or *PRDX3* deficiency in vivo promoted liver fibrosis in schistosomiasis by increasing the level of hepatic ROS. These findings suggest that downregulation of *miR-383-5p* after schistosome infection may alleviate liver inflammation by de-repressing *PRDX3*, thereby increasing ROS scavenging and reducing oxidative stress. Our study elucidates the role of the *miR-383-5p*/PRDX3 axis in schistosomiasis-induced liver fibrosis and identifies PRDX3 as a promising therapeutic target for this disease.

## Data Availability

No datasets were generated or analysed during the current study.

## Abbreviations

PRDX3: Peroxiredoxin-3
ROS: Reactive oxygen species
PZQ: Praziquantel
H_2_O_2_: Hydrogen peroxide
ECL: Enhanced chemiluminescence
α-SMA: α-Smooth muscle actin
